# The Automatic Methods Group *Newsletter*

**DOI:** 10.1155/S1463924697000096

**Published:** 1997


Journal of Automatic Chemistry, Vol. 19, No. 2 (March–April 1997) pp. 55–60

AUTOMATIC METHODS GROUP

Analytical Division
ROYAL SOCIETY OF CHEMISTRY

### The Automatic Methods Group *Newsletter*

The AMG Newsletter is published as part of and in collaboration with the *Journal of Automatic Chemistry*. Further information on the *Journal of Automatic Chemistry* can be obtained from Taylor & Francis Ltd, Rankine Road, Basingstoke, Hampshire RG24 8PR or e-mail:info@tandf.co.uk; worldwide web home page: http:||tandf.co.uk.

### Meeting Reports

### *Thermal desorption—15 years on*

A joint meeting of the Automatic Methods Group and the South East Region of the Royal Society of Chemistry, in co-operation with the Health and Safety Executive and sponsored by Perkin Elmer Ltd was held on Tuesday 17 December 1996 at the Scientific Societies Lecture Theatre, London. The texts of the papers presented follow.

### Abstracts of papers presented

### Thermal desorption, the real story

**Richard H. Brown**
*Environmental Measurement Group, Health and Safety Laboratory (HSL), Health and Safety Executive, Broad Lane, Sheffield, UK*

Nearly 20 years ago, a number of laboratories specializing in monitoring volatile organic compounds VOC(s) in workplace air began to get interested in thermal desorption. This interest was initially to provide an alternative to the pumped charcoal tube/solvent desorption method, which for many compounds lacked sensitivity or had poor desorption efficiency.

However, thermal desorption had its own problems, one of which was that there was no back-up section to check on breakthrough. Early studies at HSL therefore centred on predicting safe sampling volumes, so that only one tube was needed. This work resulted in one of the most cited publications in the field by Brown and Purnell [1], which originally formed the basis of a lecture at a Chemical Society meeting in London in December 1977.

A second problem was the availability of a reliable thermal desorption apparatus, but through the agency of an HSE committee, 'Working Group 5', set up following the London meeting, Perkin-Elmer were invited to design such a desorber. This invitation resulted in the ATD-50, the first fully automated thermal desorber, in 1981, which was updated to the ATD-400, also to WG5's specification, in 1990.

The third problem was not one of thermal desorption as such, but of the sampling method. The original thermal desorption tube method used a sampling pump to collect the sample. At much the same time as WG5 was thinking about thermal desorption, the group was also thinking about diffusive sampling as a way of getting rid of the pump. It is rumoured that the idea of using a thermal desorption tube as a diffusive sampler, simply by leaving one end open, came simultaneously to half of WG5 as they travelled south from a meeting in ICI Wilton in 1979. This resulted in a second seminal paper by Brown, Charlton and Saunders [2], and the Symposium on Diffusive Sampling in Luxembourg in 1986.

The fourth problem was a matter of what sorbent to choose. In the charcoal tube method, one sorbent (coconut shell activated carbon) sufficed for a wide range of VOCs, although several different more esoteric sorbents have to be used in special cases if high desorption efficiency is to be obtained. For thermal desorption, Tenax is usually the sorbent of choice, but it has limited capacity for more volatile and polar compounds, which are often the ones of interest. Some alternative sorbents were examined in a project sponsored by the EC (MAT1-CT92-0038) in 1994/95, with the recommendation that Chromosorb 106 be used for the more volatile compounds, and Carboxen 569 for extremely volatile compounds such as methanol, propane and ethylene oxide.

A fifth problem was the availability of reference materials. Acceptable results nowadays have to be traceable to national standards, and so, in 1991, reference material was produced by the then BCR of known amounts of benzene, toluene and xylene on a Tenax tube. Quality

0142-0453/97 $12·00 © 1997 Taylor & Francis Ltd control schemes such as the HSL WASP scheme have also been set up to include thermal desorption samples.

Finally, there has been a reluctance on the part of analysts to use thermal desorption techniques unless standard methods existed, and this has led to a series of performance standards covering both thermal desorption and diffusive sampling issued by CEN, national standard methods issued by HSE and ASTM, for example, and international standards being developed in ISO.

### References

Brown, R. H. and Purnell, C. J., *Journal of Chromatography*, **178** (1979).

Brown, R. H., Charlton, J. and Saunders, K. J., *American Industrial Hygiene Association Journal*, **42** (1981).

### Thermal desorption techniques for sampling and analysis of VOCs

**Alan Braithwaite**

*Analytical Chemistry, Department of Chemistry and Physics, Nottingham Trent University, Clifton Lane, Nottingham NG11 8NS, UK*

Thermal desorption (TD) is a well-established technique for the collection and pre-concentration of organic volatiles present in ambient and workplace air and VOC emissions. TD is also used as a flexible sample introduction technique for GC, GC-MS. The aim of this paper was to outline the specification of a TD-GC system and to look at sample collection using pumped sampling.

VOCs levels in air range from approximately 0.5 μg $m^{-3}$ (0.1 ppbv) to more than 500+ mg $m^{-3}$ (500+ ppm), yet GC capillary columns require samples in the ng/100 μl range. Sorption onto polymeric sorbents with subsequent thermal desorption offers a clean efficient sample focusing method with none of the disadvantages of solvent extraction techniques. Sorbents such as the Porapak range and then Tenax and the Chromosorbs were developed in the 1960s and were soon used as GC stationary phases for quantitative routine analyses. Sorption properties depend on the surface area (30–800 $m^2$ $g^{-1}$) and macro pores present (300–10 nm) and vary depending on the monomers used to manufacture the polymers and degree of cross linking. Sampling tubes packed with a suitable polymer are effective sample collection units and the samples are recovered with >98% efficiency by heating the tubes to 150–300°, reducing the retention properties allowing the sample to be transferred to a GC.

Thermal desorption tubes are often thought of as short GC columns. Whilst this approach is valid for predicting some of the theoretical aspects of sorption-desorption in a dynamic (pumped) sampling system in reality the sampling process is similar to displacement chromatography. Sorbents, sampling flow rate and sample time are selected so that the VOCs in an appropriate volume of air are efficiently collected without migrating to the other end of the tube. Break-through volumes for a wide range of VOCs have been determined for a number of sorbents so that a 'safe' volume of air is collected and the safe sampling volume is not exceeded. Since the sorption-desorption process is dependent on vapour pressure of the individual analytes, the effect of changes in temperature at which the samples are taken can be a problem unless taken into account and the sampled volume adjusted accordingly. Rapid desorption at 150°+ and sample focusing followed by capillary column GC gives a chromatogram from which qualitative and quantitative data can be obtained. The aim of this paper is to explain sorption-desorption, and sampling criteria relevant to TD-GC.

### Diffusive sampling and thermal desorption: an odd couple?

**Theo Hafkenscheid**

*NMi Van Swinden Laboratory, Delft, The Netherlands*

The development of diffusive sampling into an accepted alternative for the traditional pumped sampling for the determination of gases and vapours in air almost parallels the development of thermal desorption as an alternative for solvent desorption. Diffusive sampling is now applied for measurements in all types of air, workplace as well as indoor and ambient. Diffusive sampling combines some inherent advantages (simplicity, unattended operation, economy), with disadvantages. The major disadvantage is the impossibility of demonstrating proper functioning 'on the spot'.

The combination of diffusive sampling and thermal desorption adds advantages and disadvantages. The key factor is the selection of an optimum sorbent, which should combine sometimes irreconcilable properties, such as strong sorption, high temperature stability and low blank development. The (sometimes unavoidable) application of 'compromise' sorbents leads to deviations of the sampler uptake from ideal behaviour due to sorbent saturation or back diffusion.

The process of selection, testing and validation of a thermally desorbable diffusive sampler is therefore not paramount to the successful performance of a sampler, but also represents an often intricate and challenging procedure.

This presentation focused on some of the possible approaches to the selection and testing of sorbents, and explained their merits and drawbacks.

### EU ambient air directive: impact and implementation

**Annette Borowiak and Emile De Saeger**

*European Commission, Joint Research Centre, Environment Institute, European Reference Laboratory of Air Pollution, I 21020 Ispra (VA), Italy*

The European Commission has proposed a new Framework Directive on ambient air quality assessment and management, and this was adopted by the European Council in October 1996. Implementing the existing directives involved several problems, such as poor data comparability, long procedures to prepare directives for new pollutants and the poor respect of guide values. The general aim of the new directive is to define the basic principles of a common strategy to: define and establish objectives for ambient air quality; assess the ambient air quality on the basis of common methods and criteria; obtain information on ambient air quality and make it available to the public; and maintain ambient air quality where it is good and improve it in other cases.

The details of each pollutant are dealt with in specific daughter directives. Air quality monitoring has a special place in the framework directive. The directive makes provisions for network design and site selection criteria, data quality objectives, reference measurement methods and the implementation of standardized quality systems. These provisions will allow the use of screening methods based on diffusive sampling technique as a tool for the siting of network stations, for preliminary assessments of ambient air quality, for monitoring in areas not exceeding limit values and for the classification of zones.

The list of substances to be covered by the framework directive concerns the pollutants already considered in existing ambient air quality directives ($SO_2$, $NO_2$, PM 10/SPM, Pb, $O_3$) and new air pollutants, for example benzene, PAH, CO and heavy metals (Cd, Ni, As, Hg).

### Measurement quality—how not to do it!

**Bill Boyle**

*BP International, Sunbury on Thames, Middlesex TW16 7LN, UK*

Quality control and assurance are key to the success of workplace, ambient or indoor air monitoring surveys when using sorbent tubes and thermal desorption. The importance of quality procedures for both sampling and analysis were discussed in this paper.

The level of quality, in terms of precision and bias, or repeatability and reproducibility, should be established in terms of the objectives of a monitoring survey. Internal quality control and external quality assurance are used to achieve satisfactory levels of overall uncertainty for comparison of results with limit values. The recent standard EN482, and the proposed equivalent for ambient air, provide a basis for assessing methods. The implementation of a quality system and achievement of accreditation under national schemes provides a degree of confidence in laboratory data. However, participation in recognized quality assurance schemes, if available, provides a more robust approach and increases confidence in the precision of the results.

Laboratory measurements, particularly at low parts per billion levels, are only ever as good as the sampling procedure used. Extending the quality principles to the field is necessary to ensure that a measurement technique is truly valid. Good sampling practices, chain of custody documentation and training are all aspects which need to be considered.

The current status of QA/QC tools were reviewed in the paper and illustrated with practical examples on field sampling and laboratory analysis pitfalls.

### ATD as an autosampler

**Elizabeth Woolfenden**

*Perkin-Elmer, USA*

Many industrial QA/QC and materials research applications require measurements of volatile and semi-volatile organics. Capillary gas chromatography is the ideal analytical tool for these compounds, but it is not directly compatible with most real-life samples. Multi-step solvent extractions and even steam distillation are often required before a sample can be injected into a gas chromatograph. These sample preparation procedures suffer many disadvantages, for example high risk of error introduction, poor quantitative performance, labour/cost-intensive, masking of peaks of interest with the solvent, and analyte dilution, etc.

Automated thermal desorption (ATD) can provide a labour-saving, automatic alternative to conventional manual sample preparation. It allows quantitative gas extraction of analytes from the sample matrix and gas phase transfer (injection) of the components of interest directly into a GC analytical column. The approach is applicable to sample matrices as diverse as plastics, creams, powdered drugs, dried foods, resins, liquid suspensions, salt solutions, soap and printed packaging. In these cases the ATD becomes an automatic sample preparation device and an automatic GC injector, rolled into one.

In some cases, sample inhomogeneity and/or high water content mean that a preliminary off-line gas extraction step is required before thermal desorption. This technique is conventionally referred to as 'purge and trap' and can also be automated. However, most materials are compatible with direct thermal desorption/extraction. Details of how to select the best approach for a range of common sample types and practical guidance on method optimization were summarized in this paper. Example data and chromatographic results were presented.

### Purge-and-trap techniques, and applications using ATD

**Alexander P. Bianchi**

*Exxon Chemical Co., Fawley, Surrey, UK*

Purge-and-trap (P&T) headspace analysis (otherwise known as dynamic 'non-quilibrium' headspace analysis) is a powerful and sensitive analytical technique for the determination of a wide range of non-polar (and some polar) *purgeable* volatile organic compounds (VOCs) present at very low concentrations ($< 0.1\ \mu g\ l^{-1}$) in water and interstitial waters within sediments.

Since its development in the early 1970s by Grob and coworkers, the method has undergone many refinements. Fundamentally, P&T analysis involves purging a purified inert gas such as helium *through* the body of water or sediment slurry. The experimental protocol requires that the sample is held at a steadily controlled temperature, and purged at a constant rate over a fixed time period while the purge gas continuously strips VOC from the sample medium.

Individual VOCs are purged at different rates (and efficiencies) depending on physicochemical factors such as their aqueous solubilities, n-octanol/water partition coefficients ($\log_{10} K_{ow}$), vapour pressures (i.e. Henry's Law constants) and the stripping flow rate and temperature. The number, size and flowpath geometry of the finely dispersed gas bubbles is also important to maximize contact area and contact time with the sample.

The purging gas is passed out of the stripping vessel and through an adsorbent bed, itself inert to the gas, where the purged VOCs are selectively trapped in the body of the adsorbent media (for example Perkin-Elmer ATD50 or ATD400 tubes packed with Tenax-TA, Chromosorb-106, Spherocarb, or many of the new Supelco Inc. carbon molecular sieves which are increasingly being used). In experiments designed to assess the comparative performance of Perkin-Elmer tubes packed with multi-sorbent beds and analysed by ATD thermal desorption versus activated charcoal tubes which were desorbed with carbon disulphide, the former approach yielded significantly superior method performance in both experimental and field work trials.

The P&T approach described here is based on the Open-Loop Stripping Analysis (OLSA) technique which forms the basis of many USEPA methods for pollution assessment. The basic method has been successfully and broadly applied to potable water, river water, wastewater and marine environmental studies of VOC, including the examination of toxicologically significant compounds and biogenically-derived species at ppb concentration levels. The method is particularly amenable to the study of volatile aromatics, alkanes, alkenes, organohalogens and organosulphides. Oxygenated volatiles such as aldehydes, ketones and alcohols may also be determined under closely controlled conditions.

Application of the OLSA-P&T method with ATD5O and ATD4OO thermal desorption has been used successfully to measure VOC from volatile end products in water chlorination processes to volatile bio-products arising from phytoplankton blooms in coastal seawater.

### Chemotaxonomy of plants using headspace and ATD

**J. D. Twibell**

*NCCPG Artemisia Collection, Elsworth Herbs, Cambridge, UK*

Scented plants produce volatile oils which consist of numerous individual organic components. The particular blend of components gives rise to the characteristic odour of the species or variant. Amongst many species within the *Artemisia* genus there are a number of regional variants which can be readily segregated by odour differences. Analysis of the vapours from such plants enables the subtle differences to be exploited and recorded and the characteristic vapour profiles form a basis for chemotaxonomy.

While some species can be separated and identified into subgroups or regional variants by this technique, other species appear more stable and show little variation across their geographical distribution. Although the overall vapour profiles for a given variant remain similar throughout the growing season, changes do appear to occur in the relative ratios of some components. To avoid this, and other potential false discrimination problems, plants should ideally be grown in the same location and should be sampled at the same time of year.

Some individual components of plant vapours are now known to influence the growth or predator response of other plants even of different genera and the headspace ATD-MS method may be useful as a means of screening plants for potential phytoactive volatiles. The methods of sampling are discussed in this paper.

### Acknowledgement

The author gratefully acknowledges a grant from the National Council for the Conservation of Plants and Gardens (NCCPG) for the use of analytical equipment.

### On-line ATD to monitor ozone precursors

**Greg Johnson**

*Perkin-Elmer, Beaconsfield, UK*

Volatile Organic Compounds (VOCs) are of national and international concern and, increasingly, are the subject to legislation. Certain VOCs are believed to contribute to the depletion of stratospheric ozone and mechanisms that influence global warming. Some VOCs are believed to be toxic or carcinogenic, while some are known to contribute to the formation of ground level ozone and photochemical smog in urban and industrial areas.

In the lower atmosphere, ozone can be formed as a result of the photo-dissociation of nitrogen dioxide ($NO_2$) to form nitrogen oxide (NO) and atomic oxygen. Nitrogen oxide reacts rapidly with ozone to regenerate $NO_2$ and therefore, a steady state ozone concentration is obtained. The presence of certain, reactive (ozone precursors) VOCs, creates a reaction pathway that allows NO to be converted back to $NO_2$, without consuming a molecule of ozone, thus allowing the latter to accumulate. Ozone is therefore described as a secondary pollutant and is a reactive gas that can be harmful to living tissue.

Several methods for the determination of atmospheric VOCs have been reported and these include on-line systems for continuous measurement. In some cases these systems have had a restricted volatility range. Others, including commercially available analysers, require large quantities of liquid cryogen, that increase running costs and may restrict the options concerning the siting of such a monitor.

The system described in this paper is composed of two key functional units:

(1) Quantitative analysis of VOCs requires a system capable of achieving detection limits below the part per billion level. Sample analysis is required on an hourly basis and sample collection should be of sufficient duration, as to be representative of this time interval. The system described here utilizes an electrically powered, Peltier cooled adsorbent trap to achieve these requirements. A liquid cryogen is not required.

(2) The sample is of a complex nature and severely stretches the capability of any chromatographic system that is based on a single column. The system described uses a novel Deans switch to perform a multidimensional separation of the complex mixture. In effect, sample components are selectively distributed to the columns most appropriate for the analysis of a specific volatility range. This provides an extremely flexible system capable of adaptation, should regulatory requirements alter in the future.

### A novel device for capturing alveolar breath samples for solvent analysis

**D. Dyne, J. Cocker and H. K. Wilson**
*Health and Safety Laboratory, Broad Lane, Sheffield S3 7HQ, UK*

For many solvents used in industry, biological monitoring methods require the analysis of the solvent in blood as an assessment of exposure. However, the collection of blood samples is often not welcomed by the workers. A non-invasive alternative is the measurement of solvents in alveolar air as an indicator of exposure.

The authors have developed a novel breath sampling device suitable for capturing a portion of alveolar air. This breath is then transferred onto a diffusive sampling tube which is subsequently analysed by automated thermal desorption-gas chromatography-mass spectrometry. The detection limit of the method is 1 nmol/l. Intra- and inter-assay variation are 4.85 and 9.6% respectively. Calibration curves are prepared using methanolic solutions of solvents.

The breath sampler has been used to assess exposure to solvents in several industries, including the shoe manufacturing industry, the inks and coatings industry and at dry cleaning establishments. Solvents detected include ethyl acetate (6.4–25.5 nmol/l), propan-2-ol (3.4–39.3 nmol/l), 2-butanone (0–6.6 nmol/l) and tetrachloroethene (0–557 nmol/l).

The breath sampler has also been used to monitor excretion of solvents from human volunteers after controlled atmosphere chamber studies. More than 500 breath samples have been analysed from 24 volunteers in exposures to nine different solvents (toluene, trimethyl benzene, tetrachloroethene, tetrahydrofuran, acetone, propan-2-ol, xylene, 2-butanone, 1-methoxy 2-propanol and n-hexane). Solvent concentrations ranged from 20 ppm (n-hexane) to 400 ppm (propan-2-ol) and the duration of exposures varied from 2 hours (toluene, tetrachloroethene, propan-2-ol, 2-butanone, 1-methoxy-2-propanol and n-hexane) to 12 hours (xylene). Breath samples obtained during and after exposure ranged from 0–1,755 nmol solvent/l breath (butanone). The patterns of elimination of the solvents from breath were studied.

### Forthcoming conferences

### Petroanalysis 97

### To be held at BP Oil and Technology Centre, Sunbury on Thames on Wednesday 21 May 1997.

The conference should be considered a follow-up to the last very successful Petroanalysis conference of 1988. The aim of Petroanalysis 97 is to bring analysis in the oil, petrochemical and related industries up-to-date. The conference will comprise a series of presentations looking at automation, online analysis, modern analytical techniques and the problems associated with a 'downsizing' of analytical facilities.

#### Programme

Morning Session: Chairman, Dr Harry Read, BP Oil and Technology Centre, Sunbury

| | |
|---|---|
| 10.30 | Petroanalysis, where are we now?<br>Dr Chris Bartlett, DRA, Farnborough |
| 11.00 | Automated determination of base and acid numbers<br>Professor Malcolm Fox, Department of Chemistry, De Monfort University, Leicester |
| 11.30 | Microwave spectrometry applied to the petrochemical industries<br>Professor Fred Alder, DIAS, UMIST, Manchester |
| 12.00 | Recent developments in gasoline analysis using IR spectroscopy<br>Dr Michael Croudace, PetroSpec Inc., Tuftin, California |

Afternoon Session: Chairman, Dr Bob Hooks, Shell Research and Technology Centre, Thornton

| | |
|---|---|
| 13.30 | Test houses for the next millennium<br>Paul Salter, Fuchs Lubricants, Hanley |
| 13.55 | Inter centre precision monitoring schemes<br>Dr Harry Read, BP Oil and Technology Centre, Sunbury |
| 14.20 | Surface studies of oil seal degradation<br>Dr Rod Davies and Dr G. Smith, Shell Research and Technology Centre, Thornton |
| 15.10 | Elemental analysis by X-ray fluorescence<br>Mr Keith Field, Oxford Instruments, Abingdon |
| 15.35 | Automated instrumentation for on-ship monitoring<br>Mr Chris Lee Jones, Kittewake Ltd, Littlehampton |

Fur further details please contact Dr R. Narayanaswamy at DIAS, UMIST, PO Box 88, Manchester M60 1QD; Tel: 0161 200 4891/4885; fax: 0161 200 4881/4911; e-mail: ramier.narayanaswamy@umist.ac.uk.

### The International LIMS Conferences and the Automatic Methods Group

**Alan S. McLelland**
*Institute of Biochemistry, Royal Infirmary, Glasgow G4 0SF*

There can be few analytical chemists who are not aware of the series of International LIMS Conferences which have been running annually since 1987. Such is the current importance of IT, particularly in relation to the ever-increasing list of regulatory requirements which afflict the analytical laboratory, that the LIMS conferences from the outset found a receptive audience among scientists, technologists, and laboratory managers eager to debate the implications of this powerful technology for their business. What is less well-known, however, is the role played by the Automatic Methods Group in the genesis of the International LIMS Conferences, and in the European Conferences in particular.

Gerst Gibbon, from the Federal Energy Technology Centre in Pittsburgh, who chaired the first US conference, and has been an ever-present driving force within the US organising committee since then, traces the source of the LIMS Conferences to a meeting between himself, Dave Nelson and Harmon Brown of Nelson Analytical, and Graham Martin of ICI in 1985 [1]. That meeting stemmed from the first of AMG's Ferndown meetings at the Dormy Hotel, where Gerst first met Harmon Brown, Graham Martin, Ken Leiper and Derrick Porter, all of whom were to become members of the organising committee for the first LIMS Conference.

A second meeting over a working breakfast at Pittcon in Atlantic City in March 1986, which again featured Gerst, Harmon, Graham, Ken and Derrick, among others, brought unanimous agreement that there should be a series of International LIMS Conferences alternating between the USA and Europe. In practice, the first two were held in Pittsburgh in 1987 and 1988.

The LIMS/AMG links were further reinforced when Gerst was asked to present a keynote lecture 'LIMS—what are the choices? at the Ferndown meeting 'Analytical Chemistry—a time for change?' in October 1987. Other AMG luminaries present were Doug Squirrell, Derrick Porter, Gordon Farrow, Ken Leiper, Alan Braithwaite and the AMG chairman, then, as now, Kevin Saunders.

The first European Conference (3rd in the series) was held in the UK in 1989, at the Anugraha Conference Centre, under Graham Martin's chairmanship. From the outset, it was agreed that the European conference committee would function as a sub-group of the Royal Society of Chemistry's Automatic Methods Group. The LIMS Conference was therefore allowed to use the RSC's crest on its publicity material, and the intellectual cachet thus afforded the fledgeling conference was very much appreciated. The AMG also kindly offered a starter grant towards the 3rd conference, which was repaid in full from the profits of the meeting.

AMG Treasurer Alan Braithwaite has been Honorary Treasurer of the European Conferences since their inception, and all European LIMS Conference accounts are kept within the RSC, although the deregulation of the RSC for VAT caused a few headaches for the 7th Conference in 1993.

As the European Conferences gathered pace, the chairmen were routinely appointed from within the ranks of the AMG—John Boother (5th Conference); Alan McLelland (7th), and Alex Williams (9th) oversaw the gradual transition of the conference to a truly European event, marked by the selection of a European mainland venue (Maritim Hotel, Bonn) for the 9th conference in 1995.

The 11th Conference, under the chairmanship of John Trigg of Kodak UK, will be held in June this year, at another new European mainland venue (The Netherlands Congress Centre, The Hague) in association with the 10th anniversary of the founding of the Division of Computational Chemistry of the Royal Netherlands Chemical Society, the Koninklijke Nederlandse Chemische Vereniging. This association with the RSC's counterpart organisation in the host country continues to be of inestimable value in assisting the success of the meeting, and, no less importantly, in fostering links between chemists world-wide.

What of the future? Astonishingly perhaps, the International LIMS Conference concept shows no sign of flagging. Ten years from inception, this year's meeting in The Hague will be the largest of its kind ever held in Europe with over 500 sq m of exhibition and a packed 4-day programme featuring short courses, plenary lectures, breakout sessions and manufacturer workshops. As the LIMS concept has matured and changed over the past decade, so the conference has kept pace, for example by integrating scientific computing into its programme.

The conference committee has supported LIMS meetings in Europe in the off-year when the main conference is in the USA, and is actively looking at proposals both for 1998 and for another new European venue for the 13th conference in 1999. Like the venues, the organising committee is gradually becoming more mainland-oriented with German, Norwegian, Belgian and French representation, and the chairman-elect for 1999 is Professor Reinhold Schaefer of the Technical University of Weisbaden. Nevertheless, for the foreseeable future, the European International LIMS Conferences will continue to be associated with, and promote the values of, the Royal Society of Chemistry, through its continuing and valued links with the RSC's Automatic Methods Group.

### References

1. Gibbon, G.A., *A brief history of LIMS.* Laboratory Automation and Information Management, (1996), **32**, 1–5.

*For further information about the 1997 LIMS Conference, contact the Conference Secretariat LIMS 97, 45 Hilltop Avenue, Hullbridge, Hockley, Essex, SS5 6BL, UK. Tel.: +44 1702 231268; fax: +44 1702 230580; e-mail 101320.1671@compuserve.com or see conference updates on the LIMS97 website: URL: http://www.LIMS97.com. To submit a paper or poster, contact Siri Segalstad at Segalstad Consulting, P.O. Box 15, Kjelsas, N-0411 Oslo, Norway. e-mail Segalst@powertech.no*

### Obtaining information about the AMG

Readers are invited to look at the Automatic Methods Group's pages available on the Internet at http://chemistry.rsc.org/rsc/amg.htm or alternatively via the RSC's home page at http://chemistry.rsc.org/rsc/. Information to be found at these sites includes: details about the AMG committee, the group's objectives, a programme of forthcoming meetings, and reports on meetings that have already taken place, as well as the most recent edition of the AMG Newsletter.

The AMG also plan to communicate information about future meetings. Anyone interested in receiving information by e-mail should send an e-mail to the Hon. Secretary of the Group, Derrick Porter, at dgporter@-argonet.co.uk.

